# A focus groups study on data sharing and research data management

**DOI:** 10.1038/s41597-022-01428-w

**Published:** 2022-06-17

**Authors:** Devan Ray Donaldson, Joshua Wolfgang Koepke

**Affiliations:** Department of Information and Library Science, Luddy School of Informatics, Computing, and Engineering, Indiana University, Bloomington, Indiana US

**Keywords:** Research data, Research management

## Abstract

Data sharing can accelerate scientific discovery while increasing return on investment beyond the researcher or group that produced them. Data repositories enable data sharing and preservation over the long term, but little is known about scientists’ perceptions of them and their perspectives on data management and sharing practices. Using focus groups with scientists from five disciplines (atmospheric and earth science, computer science, chemistry, ecology, and neuroscience), we asked questions about data management to lead into a discussion of what features they think are necessary to include in data repository systems and services to help them implement the data sharing and preservation parts of their data management plans. Participants identified metadata quality control and training as problem areas in data management. Additionally, participants discussed several desired repository features, including: metadata control, data traceability, security, stable infrastructure, and data use restrictions. We present their desired repository features as a rubric for the research community to encourage repository utilization. Future directions for research are discussed.

## Introduction

Sharing scientific research data has many benefits. Data sharing produces stronger initial publication data by allowing peer review and validation of datasets and methods prior to publication^1,2^. Enabling such activities enhances the integrity of research data and promotes transparency^1,2^, both of which are critical for increasing confidence in science^3,4^. After publication, data sharing encourages further scientific inquiry and advancements by making data available for other scientists to explore and build upon^2–5^. Open data allows further scientific inquiry without the costs associated with new data creation^4,6^. Researchers in the developing world disproportionately experience the high costs of new data creation as they may struggle to find funding for projects not associated with direct improvement in living conditions^6^. Therefore, data sharing can enable lower-cost research opportunities within developing nations through reusing datasets, creating what Ukwoma and Dike^7^ refer to as a “global network” of scientific data. The development of vaccines for the COVID-19 virus illustrates the impact of data sharing on society. Through open data sharing practices, including sharing the genome sequence for the virus, scientists were able to build on each other’s data to create vaccines in record time^8,9^, saving millions of people’s lives.

Despite the benefits, research has shown that many scientists still do not share their research data^10,11^. Most disciplines operate without established data sharing or data management guidelines, relying on individual or institutional solutions for data management and sharing^10^. Exceptions include research associated with government funding, specific grant restrictions, or approval requirements from institutional review boards (IRBs).

Current scholarship on data management and data sharing within academic disciplines is fragmented. Few interdisciplinary studies exist, and of these, important topics, such as data librarianship and scientists’ perceptions of necessary repository features, are left out of analysis^12–15^. Also, singular discipline studies on scientific data management and repository utilization contribute limited views and are dated^16–18^.

Applying the conceptual framework of Knowledge Infrastructures (KI) provides a basis for understanding the creation, flow, and maintenance of knowledge^19^. KI posits that seven interconnected entities account for the system: shared norms and values, artifacts, people, institutions, policies, routines and practices, and technology^19^. Examination of some or all of these entities throw into relief inefficiencies and areas for growth within knowledge creation, sharing, and maintenance. In particular, prior research^11^ points to repositories and human resources as key areas of investment to improve data management and increase sharing within the scientific community.

This study uses KI as a lens to focus on the individual scientist (i.e., people), their use of repositories (i.e., routines and practices/technology), their opinions of librarians and data sharing (i.e., norms and values), and their data management plans (i.e., policies/institutions). We explore the data management and sharing practices of scientists across five disciplines by answering the following research question: what features do scientists think are necessary to include in data repository systems and services to help them implement the data sharing and preservation parts of their data management plans (DMPs)? We found a consensus across disciplines on certain desired repository features and areas where scientists felt they needed help with data management. In contrast, we found that some discipline-specific issues require unique data management and repository usage. Also, there was little consensus among study participants on the perceived role of librarians in scientific data management.

This paper discusses the following. First, we provide an analysis of the results of our focus group research. Second, we discuss how our study advances research on understanding scientists’ perspectives on data sharing, data management, and repository needs and introduce a rubric for determining data repository appropriateness. Our rubric contributes to research on KI by providing an aid for scientists in selecting data repositories. Finally, we discuss our methods and research design.

## Results

### Scientists’ data practices

Participants across all the focus groups indicated having a DMP for at least one of their recent or current projects. Regarding data storage, some participants across four focus groups (atmosphere and earth science, chemistry, computer science, and neuroscience) used institutional repositories (IRs) for their data at some point within the data lifecycle, with five participants explicitly indicating use of IRs in their DMPs. The other popular choice discussed across four focus groups (atmospheric and earth science, computer science, ecology, and neuroscience) was proprietary cloud storage systems (e.g., DropBox, GitHub, and Google Drive). These users were concerned about file size limitations, costs, long-term preservation, data mining by the service providers, and the number of storage solutions becoming burdensome.

### Desired repository features

#### Data traceability

Participants across four focus groups (atmosphere and earth science, chemistry, ecology, and neuroscience) mentioned wanting different kinds of information about how their data were being used to be tracked after data deposit in repositories. They wanted to know how many researchers view, cite, and publish based on the data they deposit. Additionally, participants wanted repositories to track any changes to their data post-deposit. For example, they suggested the creation of a path for updates to items in repositories after initial submission. They also wanted repositories to allow explicit versioning of their materials to clearly inform users of changes to materials over time. Relatedly, participants wanted repositories to provide notification systems for data depositors and users to know when new versions or derivative works based on their data become available as well as notifications for depositors about when their data has been viewed, cited, or included in a publication.

#### Metadata

Participants across three focus groups (atmospheric and earth science, chemistry, and neuroscience) discussed wanting high quality metadata within repositories. Some argued for automated metadata creation when uploading their data into repositories to save time and provide at least some level of description of their data (e.g., P1, P4, Chemistry). Within their own projects and in utilizing repositories, participants wanted help with metadata quality control issues. Participants within atmospheric and earth science who frequently created or interacted with complex files wanted expanded types of metadata (e.g., greater spatial metadata for geographic information system (GIS) data). Atmospheric and earth scientists, chemists, and neuroscientists wanted greater searchability and machine readability of data and entities within datasets housed in repositories, specifically to find a variable by multiple search parameters.

#### Data use restrictions

Participants across all five focus groups agreed that repositories need to clearly explain what a researcher can and cannot do with a dataset. For example, participants thought repositories *should clearly state on every dataset* whether researchers can: base new research on the data, publish based on the data, and use the data for business purposes. Participants stated current data restrictions can be confusing to those not acquainted with legal principles. For example, one data professional (P2, Chemistry) explained that researchers often mislabeled their datasets with ill-suited licenses. Participants commonly reported using Open Access or Creative Commons, but articulated the necessity of having the option for restrictive or proprietary licenses, although most had not used such licenses.

Some participants used embargoes and others never had. Most viewed embargoes as “a necessary evil,” provided that they are limited to approximately a few years after repository submission or until time of publication. Participants did not think it was fair to repository staff or potential data reusers to have any data embargoed in perpetuity.

#### Stable infrastructure

Participants across two focus groups (atmospheric and earth science, and chemistry) expressed concern about the long-term stability of their data in repositories. Some stated that their fear of a repository not being able to provide long-term preservation of their data led them to seek out and utilize alternative storage solutions. Others expected repositories to commit to the future of their data and have satisfactory funding structures to fulfill their stated missions. Participants described stable repository infrastructure in terms of updating data files (i.e., versioning) and formats over time and ensuring their usability.

#### Security

Participants across four focus groups (atmospheric and earth science, chemistry, computer science, and neuroscience) discussed wanting their data to be secure. They feared lax security could compromise their data. Specific to embargoed data, they feared lax security could enable “scooping” of research before data depositors are able to make use of the data through publication. Those handling data with confidential, sensitive or personally identifiable information expressed the most concern about potential security breaches because it could result in a breach and loss of trust with their current and future study participants, making it harder for themselves and future researchers to recruit study participants in the long-term, and it would result in noncompliance with mandates from their IRBs.

### Desired help with aspects of data management

#### Help with metadata standardization and quality control

Participants across four focus groups (atmospheric and earth science, chemistry, ecology, and neuroscience) discussed wanting help with metadata standardization, including quality control for metadata associated with their datasets, to help fulfill their DMPs while enhancing data searchability and discoverability.

#### Help with verification of deleted data when necessary

Our university-affiliated participants were particularly concerned about verifiable deletion of data when necessary to comply with their IRBs. Participants expressed concern about their newer graduate students’ capacity and follow-through on deleting sensitive data that their predecessors (i.e., graduate students who graduated before study completion) collected before they started school. In this scenario, failure to delete sensitive data is a serious breach of IRB policy, which can lead to the data being compromised and/or revocation of permission to conduct future research. Participants who worked with sensitive information frequently (e.g., passwords in computer science and medical information in neuroscience) cited this as a concern.

#### Need for data management training

Participants across four focus groups (atmospheric and earth science, chemistry, ecology, and neuroscience) mentioned the need for additional training in data management. Participants stated they were unaware of the number of discipline-specific repositories that were currently available until their peers or librarians shared this information. Consequently, several participants suggested training sessions to raise awareness about these repositories within their disciplines. Participants were also concerned about what they perceived as a limited amount of training available for graduate students and new researchers on data management tools. They described current efforts that they were aware of as either training new workers/students on simpler tools or conducting training “piecemeal” on advanced data management tools, both of which they perceived as limiting project productivity. No participants in the computer science focus group mentioned the need for additional technical or informational training in data management.

### Knowledge of existing data management principles and practices

Results were mixed on participants’ knowledge about the FAIR Guiding Principles for Scientific Data Management and Stewardship^20^. Twelve participants across all five focus groups knew about the FAIR principles, while ten across four disciplines (chemistry, computer science, ecology, and neuroscience) did not know about them. Those who were familiar mentioned challenges with applying the FAIR principles to large and multimodal datasets (e.g., P4, Neuroscience).

### Role of librarians in data management

Participants across two focus groups (atmospheric and earth science and chemistry) did not think librarians should have a role in their data management for two reasons. First, they thought their data were too technical or specialized for librarians to meaningfully contribute to their management (e.g., P3, Chemistry). Second, they assumed librarians were too busy to help with data management. In their view, librarians were already stretched too thin with greater priorities related to addressing the “modern era of information overload” to be concerned with managing their data (e.g., P6, Chemistry).

In contrast, other participants across all five focus groups thought librarians could play a role in scientific data management and sharing by providing assistance with publication, literature searches, patents, copyright searches, management of data mandates, embargo enforcement, information literacy, and metadata standardization. The two largest areas of agreement for participants who indicated a role for librarians were the more traditional area of assistance with information research and search help (e.g., P5, Atmospheric and Earth Science) as well as data management (e.g., P4, Neuroscience).

## Discussion

This study contributes to the research literature on scientists’ perspectives on data management, repositories, and librarians. Additionally, our study presents a rubric based on the perceived importance of repository features by our participants as a decision-support tool to enable the selection of data repositories based on scientists’ data management and sharing needs and preferences.

Our results suggest several aspects to improve KI focused on research data management and sharing. For example, in terms of data management wants and needs, participants in the atmospheric and earth science, ecology, and neuroscience disciplines who stated a need for help with metadata also wanted greater searchability of metadata from repositories. However, poor metadata searchability might be the net result of poor metadata quality control by data producers during data creation and/or deposit, which repositories can provide guidelines for, but in many cases cannot force data producers to do (or do well). This may be an example of KI entities (routines and practices impacting technology allowances of the corresponding repository) impacting each other. Metadata regulation issues are consistent with prior research^12,18,21–25^.

Data integrity challenges that researchers needed assistance with were developing data standards and the technical skills of employees. Both issues connect to data integrity and trust development, which are vital for data sharing and reuse^1,2^. The need for training on data management topics is consistent with prior research^25–27^. Within metadata standards development, this study’s results point to the discipline-specific call for repository integration of GIS data for discoverability by atmospheric and earth science participants. Sixty percent of participants within this subject area expressed a desire for such metadata additions. This study recognizes the need for standardized, more descriptive, and quality controlled metadata for repositories, highlighting the metadata problem faced by open access initiatives. Open access repositories have significant metadata issues, especially between repositories, which limit their searchability^28,29^. Future research on creating standards and corresponding guides to encourage better metadata creation by dataset originators may advance description and open access efforts.

Our findings are on trend with predictions from prior research about the policies, routines, and practices of data storage during and after scientists’ studies. For example, our finding of utilization of cloud storage solutions was predicted in prior research to increase over time^15^. Additionally, our study confirms overall low IR usage within^14^ and between disciplines^12–14^ with a slight increase in IR utilization over time^13^. Future research on DMP use and repository integration within DMPs may contribute to the KI entity of policy, possibly influencing more well-formed data, more shared data, and increased data integrity.

Applying the people aspect of KI to our data set, our participants did not have a consensus on the role of librarians in data management and sharing. To them, librarians’ roles appear largely ad hoc and dependent on individual institutions and librarians. Articulations of their roles varied broadly, including: teaching data management skills, implementing data standards, helping with legal aspects (e.g., rights management), and resolving technical preservation issues that concern scientists^30^. Interestingly, participants framed librarian roles in data management as a dichotomy between help with technical issues (e.g., programming skills) and traditional librarianship skills (e.g., literature search and journal access). Whether true or not, scientists’ assumptions about librarians’ roles likely have an impact on their utilization of librarians and libraries for data management and sharing. Exploring scientists’ perceptions of librarians’ roles may provide the necessary insight to foreground collaboration between scientists and librarians on, for example, improving dataset integrity^31^ and increasing dataset usage^32^, helping to justify the costs associated with making data open.

Finally, examining the routines, practices, norms, and technologies used by researchers regarding repositories has brought to the surface both an appreciation of open data as a concept and a lack of provision of open data by some of the same scientists who think open data is a good idea. A reluctance to provide open data may stem from the perceived lack of repository features discussed above, in the section Desired Repository Features, in whatever repositories scientists may have entertained using at some point in the past. Consequently, in an effort to encourage greater and more effective repository utilization by scientists across disciplines, we present a repository evaluation rubric based on the desired repository features for which we found empirical support in our study (see Supplementary Table 1).

The intended users of this rubric are scientists, librarians, and repository managers. Scientists can use our rubric to compare the relative merits of repositories based on their needs and consider what features they deem important. Librarians providing data consultations may utilize our rubric when helping their patrons. Repository managers can use our rubric to evaluate their repositories and services as a decision-support tool for what areas to improve, including what features to add to their repositories. The purpose of our rubric is to aid in repository selection and critical analysis of available repositories while encouraging repositories to provide features that we have found scientists want. As a corollary, we hope to encourage an increase in data deposit by scientists thereby increasing research opportunities to advance the studied academic disciplines^2–5^. This is particularly important for scientists in developing countries who may need to be more reliant on utilizing existing datasets for cost effectiveness^6^. Moreover our rubric’s encouragement of data deposit may increase research integrity by making the data available for experts to check^1,2^.

Future studies can compare the desired repository features that we gathered empirical support for to additional desired repository features identified through conducting comparable research with scientists from similar and different disciplines and scientists from countries with differing levels of development than those we studied here to assess the generalizability and appropriateness of our instrument.

## Methods

We produced a convenience sample for our study by browsing the participants lists of major conferences in each discipline: AGU Annual Meetings for atmospheric and earth science, American Chemical Society for chemistry, SOUPS’19 and SOUPS’20 for computer science, Society for Freshwater Science Annual Meetings for ecology, and Neuroscience’19 and Neuroscience’20 for neuroscience. From these participants lists we randomly selected individuals to receive recruitment emails from us inviting their participation in our study. Snowball sampling yielded a few additional participants, and a few other participants were obtained from informal knowledge networks in online communities (e.g., subject-specific discord groups). All professionals were vetted for credentials before inclusion (e.g., master’s and/or doctoral degrees in their disciplines).

Our study participants came from a variety of educational and workplace backgrounds. Table 1 lists our study participants by subject discipline, occupation, and affiliation. Study participant selection criteria required participants to self-identify as a scientist from one of the five subject domains under investigation and possess or be in the process of obtaining a graduate-level degree (e.g., active enrollment within a graduate program) within a particular scientific discipline. We sought individuals from separate institutional affiliations, subdiscipline research backgrounds/interests, and career stages for each scientific discipline to encourage a diversity of opinions and to ensure certain data metrics would not be artificially skewed (e.g., having multiple chemistry participants who actively work together on projects or work at the same institution were likely to answer our questions similarly). All participants currently work and reside in developed western countries. Most are from the United States, with two individuals (one from the scientific discipline of chemistry and another from neuroscience) living in developed countries in western Europe. Participants ranged from researchers primarily focused on experimenting to professors combining research with teaching responsibilities to professionals providing services or guidance to researchers (e.g., a data manager for a lab). The majority were mid-career, with a few early- and late-career. Most were affiliated with universities; however, some were government-affiliated or worked at private, for-profit enterprises.

**Table 1:** Study Participants.

Discipline	Number of Participants	Occupation	Affiliation
Atmospheric & Earth Sciences	n = 5	1 - Professional	2 - Government
		2 - Professor	3 - University
		2 - Researcher	
Chemistry	n = 8	2 - Professional	1 - Government
		4 - Professor	1 - Private Enterprise
		2 - Researcher	6 - University
Computer Science	n = 4	2 - PhD Candidate	4 - University
		1 - Professor	
		1 - Professional	
Ecology	n = 4	1 - Professor	1 - Government
		3 - Researcher	3 - University
Neuroscience	n = 4	1 - Graduate Student	1 - Government
		1 - Professor	1 - Private Enterprise
		2 - Researcher	2 - University

We conducted focus groups via zoom video-conferencing software between April and August of 2021. After introductions and collecting basic demographic information (e.g., education, work experience, research interests, etc.), we asked participants questions about their data, past and present research projects, their data management, DMPs, what aspects of data management they would like help with, whether they think libraries can help, and data sharing. We used these questions to lead into a discussion about the FAIR principles and what features they thought were necessary to include in data repository systems and services to help them implement the data sharing and preservation parts of their DMPs. Specifically, we asked participants about their expectations for: file size acceptance, licensing, embargo periods, data discoverability, and reuse. Each focus group lasted approximately an hour and a half. We gave participants $50 electronic amazon gift cards as incentives for their participation. The Indiana University Human Subjects Office approved this study (IRB Study #1907150522). Informed consent was obtained from all participants.

We transcribed the focus group recordings and analyzed the transcripts in MAXQDA, qualitative data analysis software. Afterwards, we followed the steps outlined in the literature on the analysis of focus groups data^33^: (1) familiarization, (2) generating codes, (3) constructing themes, (4) revising and (5) defining themes, and (6) producing the report, to apply thematic analysis to our dataset, which is publicly available in figshare^34^.

### Limitations

While using focus groups as a data collection methodology had many benefits for us, including the distinct advantage of unscripted interactions between participants, the ability to ask follow-up questions in the moment, and ask open-ended questions to elicit an in-depth understanding of the complex and individualized topic of scientists’ data management practices^35–37^, it also had some disadvantages. Our overall sample size was small, which may have diminished the generalizability and repeatability of our results^36^. However, it is important to note that the sizes of our focus groups are consistent with the sizes of focus groups that were conducted in prior research on similar topics^13,38^. Despite the limitations of our method, we argue that the benefits of the knowledge gained from our scientific inquiry outweigh any potential drawbacks.

## Supplementary information

Supplementary Table 1

## Data Availability

The dataset generated and analyzed during the current study is available in figshare, 10.6084/m9.figshare.19493060.v1^34^.

## Code availability

No custom code was used to generate or process the data described in this manuscript.
